# Detection of a Serum Siderophore by LC-MS/MS as a Potential Biomarker of Invasive Aspergillosis

**DOI:** 10.1371/journal.pone.0151260

**Published:** 2016-03-14

**Authors:** Cassandra S. Carroll, Lawrence N. Amankwa, Linda J. Pinto, Jeffrey D. Fuller, Margo M. Moore

**Affiliations:** 1 Department of Biological Sciences, Simon Fraser University, Burnaby, Canada, V5A 1S6; 2 Centre for Drug Research and Development, Vancouver, Canada, V6T 1Z3; 3 Provincial Laboratory for Public Health and Microbiology, Alberta Health Services, Edmonton, Canada, T6G 2R7; Lee Kong Chian School of Medicine, SINGAPORE

## Abstract

Invasive aspergillosis (IA) is a life-threatening systemic mycosis caused primarily by *Aspergillus fumigatus*. Early diagnosis of IA is based, in part, on an immunoassay for circulating fungal cell wall carbohydrate, galactomannan (GM). However, a wide range of sensitivity and specificity rates have been reported for the GM test across various patient populations. To obtain iron *in vivo*, *A*. *fumigatus* secretes the siderophore, *N*,*N'*,*N"*-triacetylfusarinine C (TAFC) and we hypothesize that TAFC may represent a possible biomarker for early detection of IA. We developed an ultra performance liquid chromatography tandem mass spectrometry (UPLC-MS/MS) method for TAFC analysis from serum, and measured TAFC in serum samples collected from patients at risk for IA. The method showed lower and upper limits of quantitation (LOQ) of 5 ng/ml and 750 ng/ml, respectively, and complete TAFC recovery from spiked serum. As proof of concept, we evaluated 76 serum samples from 58 patients with suspected IA that were investigated for the presence of GM. Fourteen serum samples obtained from 11 patients diagnosed with probable or proven IA were also analyzed for the presence of TAFC. Control sera (n = 16) were analyzed to establish a TAFC cut-off value (≥6 ng/ml). Of the 36 GM-positive samples (≥0.5 GM index) from suspected IA patients, TAFC was considered positive in 25 (69%). TAFC was also found in 28 additional GM-negative samples. TAFC was detected in 4 of the 14 samples (28%) from patients with proven/probable aspergillosis. Log-transformed TAFC and GM values from patients with proven/probable IA, healthy individuals and SLE patients showed a significant correlation with a Pearson r value of 0.77. In summary, we have developed a method for the detection of TAFC in serum that revealed this fungal product in the sera of patients at risk for invasive aspergillosis. A prospective study is warranted to determine whether this method provides improved early detection of IA.

## Introduction

Invasive aspergillosis (IA) is a life-threatening infection that affects immunosuppressed individuals including those with neutropenia, chronic granulomatous disease, acquired immune deficiency syndrome, those undergoing hematopoietic stem cell transplantation (HSCT) or solid organ transplantation, especially those with graft-versus-host disease[1–3]. The causative agent of IA is most commonly *Aspergillus fumigatus*, although other *Aspergillus* species such as *A*. *flavus*, *A*. *nidulans* and *A*. *terreus* can cause invasive infections[4–6]. Invasive infections occur when fungal conidia are inhaled, germinate and penetrate the epithelia lining of the sinuses or lungs. In some cases, the fungus may disseminate hematogenously to other organs. Despite appropriate antifungal therapy, the outcome is often fatal for many patients [7–9].

A major challenge for the management of IA is the lack of early diagnosis[10,11]. Antifungal therapy is often administered only after a prolonged febrile illness unresponsive to antibiotic therapy and confirmation of IA in many cases is made at autopsy[6]. Delays in diagnosis contribute to the morbidity and mortality rates from these infections; therefore, it is critical to identify mechanisms for the early detection of fungal growth in vivo.

Several methods are used to diagnose invasive aspergillosis including computed tomography (CT) scanning[12], microscopic or histopathologic identification of fungal hyphae in tissue specimens, or culturing the organism from the infected area. However, direct microscopic examination of the fungus in clinical samples may be difficult to achieve in seriously-ill patients in whom biopsy is inadvisable, and in processed tissue samples, harsh sample preparation procedures may fragment hyphae, making them difficult to identify[13]. Non-culture based approaches for detection of *Aspergillus* include the immunodetection of cell-wall derived galactomannan (GM) or (1→3)-β-D-glucan (BG)[14,15] or by amplifying *Aspergillus*-specific DNA[16]. The reported sensitivity and specificity for serum GM testing ranges from 48–77% and 81–100%, respectively[17–20] while sensitivity and specificity for the BG test ranges from 85–100% and 36–70%, respectively[17,21,22]. A recent report from Brasier et al. (2015) found that combining GM with a suite of host proteins improved detection of invasive aspergillosis in patients undergoing treatment for leukemia [23]. The wide range of sensitivity and specificity in these studies may be due in part to the heterogeneity in the patient populations analyzed. Immunoassays using lateral flow devices (LFD) have been developed to detect *Aspergillus*-specific compounds in serum, urine or BAL of suspected IA patients [24–26].

DNA extraction for use in PCR is often done from formalin-fixed tissue samples; however, formalin fixation may damage DNA, and prevent amplification[27]. Quantitative PCR (qPCR) can also be used to diagnose IA, especially from whole blood, serum or BAL samples. This technique relies on amplification of the inter-transcribed (ITS) ribosomal region, or the 28S or 18S rRNA genes[18,19,28]. However, due to inefficient DNA extraction procedures, the requirement for large samples volumes[29], and non-standardized approaches, it has been suggested that qPCR and PCR be used as a confirmatory test rather than an initial diagnostic test[30,31] or in combination with other approved diagnostic tests[18,19]. Due to the problems associated with all of the current approaches and the need to treat invasive fungal infections in a timely manner, new diagnostic methods for IA are needed to improve clinical outcomes.

Iron is essential for growth, and in low iron environments, such as in serum, many microorganisms secrete Fe(III)-chelating molecules called siderophores. In *A*. *fumigatus*, the hydroxamate siderophore, *N*,*N'*,*N"*-triacetylfusarinine C (TAFC) is secreted soon after conidiospore germination in iron-limited media[32]. The biosynthesis of TAFC is required for fungal germination and has been shown to be essential for virulence of *A*. *fumigatus* in a mouse model of invasive aspergillosis[33,34]. We hypothesized that TAFC would be present in the serum of patients with IA and that it could represent an early diagnostic marker of invasive aspergillosis. The purpose of our study was to establish the detection limits of TAFC in human serum using ultra high performance liquid chromatography coupled with tandem mass spectrometry (LC-MS/MS). We applied this method in a proof of concept study using serum samples from patients for which we had GM test results.

## Materials and Methods

### Samples and reagents

Serum samples were obtained from healthy individuals (n = 3) and from patients diagnosed with systemic lupus erythematosis (SLE) (n = 13) from volunteers who had signed a Simon Fraser University Informed Consent Form. The Simon Fraser Research Ethics Board specifically approved this study. Samples were de-identified before analysis to maintain anonymity. A sampling of archived serum specimens from hematology patients suspected of having invasive aspergillosis were analyzed for TAFC and GM. Other than the suspected IA diagnosis and GM value, no other clinical data was available for these patients. A total of 76 serum samples were analyzed from 58 patients. Samples were collected between January 2008 and July 2011 and were stored at -80°C until analysis. An additional set of 14 GM positive serum samples (≥0.5 GM index) were analyzed from 11 patients identified as having proven or probable aspergillosis. For these samples, the requirement for informed consent was waived by the SFU Research Ethics Board who approved the study. Samples from these patients met at least one of the criteria for proven or probable aspergillosis based in the EORTC guidelines for diagnosis[31]. The serum samples were collected between June 2014 and February 2015 and stored at -80°C until analysis. LC-MS/MS analysis of clinical specimens was carried out in a blinded fashion. Control serum for analytical optimization (male, AB positive) was purchased from Sigma-Aldrich (Ontario, Canada). Methanol and acetonitrile (LCMS grade) were purchased from EMD Science (Massachusetts, USA). Formic acid (HPLC grade) was obtained from Acros Organics (New Jersey, USA).

### Galactomannan testing

Testing for galactomannan was performed using the Platelia *Aspergillus* enzyme immunoassay (Bio-Rad Laboratories, Quebec, Canada) according to the manufacturer’s recommendations and density galactomannan index (GMI) reading of ≥ 0.5 was considered positive. The serum from SLE patients (n = 13) was analyzed in three separate pooled samples as there was insufficient volume to run GM tests on the samples individually.

### Preparation of stock solutions, calibration standards and quality control samples

TAFC was isolated from low iron cultures of *A*. *fumigatus* as previously described[35]. All stock solutions of TAFC were prepared in 0.1% formic acid in acetonitrile and subsequently diluted into serum samples. Calibration standards of TAFC spiked into serum were prepared by defrosting serum from -20°C, vortexing briefly and adding an appropriate volume into a 2 ml glass vial. Standard solutions of TAFC were spiked into serum and the vials were vortexed briefly. TAFC spiked serum samples were prepared over the range 0.5 ng/ml to 5000 ng/ml and were used to assess linearity of the method. Quality control standards were prepared by spiking TAFC standard solutions into serum over a range of 5 ng/ml to 750 ng/ml.

### Extraction of TAFC from serum

Three volumes of 0.1% formic acid in acetonitrile were added to 200 μL of serum sample in an ISOLUTE PPT+ 1 ml filter well (Biotagé, North Carolina, USA). This was incubated at room temperature for five minutes and vacuum filtered into a 96 well collection plate. The collection plate was sealed with tape and briefly agitated at low speed for one minute. Samples were concentrated by evaporating the solvent using Turbo-Vap/ N2 gas purge at 45°C. The residue was reconstituted in 200 μL of 0.1% formic acid in acetonitrile/water (50/50 v/v%) and agitated again for 1–2 minutes. This resulted in the samples being concentrated 4-fold. Five microliters of reconstituted solution was injected for LC-MS/MS analysis. Control extractions of TAFC from spiked serum samples were similarly extracted but without concentrating the extracts and used to generate a calibration curve for quantitation of TAFC in the test serum samples.

### LC-MS/MS instrument parameters and conditions

UPLC-MS/MS analysis was performed using Waters Acquity UPLC with tandem Waters Acquity PDA and TQD detectors (Waters Limited, Ontario, Canada). Separation of TAFC was done using the Waters Acquity BEH reverse phase C18 column (1.7 μm, 2.1 mm x 50 mm). Mobile phase A consisted of 0.1% formic acid in water while mobile phase B was 0.1% formic acid in acetonitrile. A gradient separation, using 10%–90% mobile phase B was performed at a flow rate of 0.3 ml/min. The injection volume was 5 μL and the column temperature was set at 30°C. The eluted TAFC was injected directly into the tandem quadrupole mass spectrometer operated in the positive electrospray ionization (ESI+) mode with a capillary voltage of 0.50 kV with nitrogen gas at a temperature of 400°C. Data was acquired in the multiple reaction monitoring (MRM) mode and Waters Empower chromatography software was used for control of the equipment and data acquisition (Waters Limited, Ontario, Canada). TAFC product ions were extracted with a span of 0.1Da.

### Statistical analyses

Each serum sample was analyzed only once because of the small volume of serum available. Quality control and calibration samples were prepared in triplicate and duplicate, respectively and analyzed individually by LC-MS/MS. Statistical analysis of the serum TAFC data in the different patient groups was performed using the Kruskal-Wallis test followed by Dunn’s post test. Logarithmically transformed TAFC and GM values were used to determine the correlation. We used the log transformation of the variables to normalize the data [36,37]. All statistical analyses were performed using GraphPad Prism software.

## Results

### LC-MS/MS method validation

The analytical method for determination of serum concentration of TAFC was reversed-phase UPLC with mass spectrometry detection. Serum concentrations of TAFC in test samples were measured against a TAFC standard calibration curve over the range 0.5 ng/ml- 5000 ng/ml. The method was evaluated for specificity, linearity, accuracy, recovery, precision, limit of detection, and limit of quantitation of the assay.

**Fig 1:**
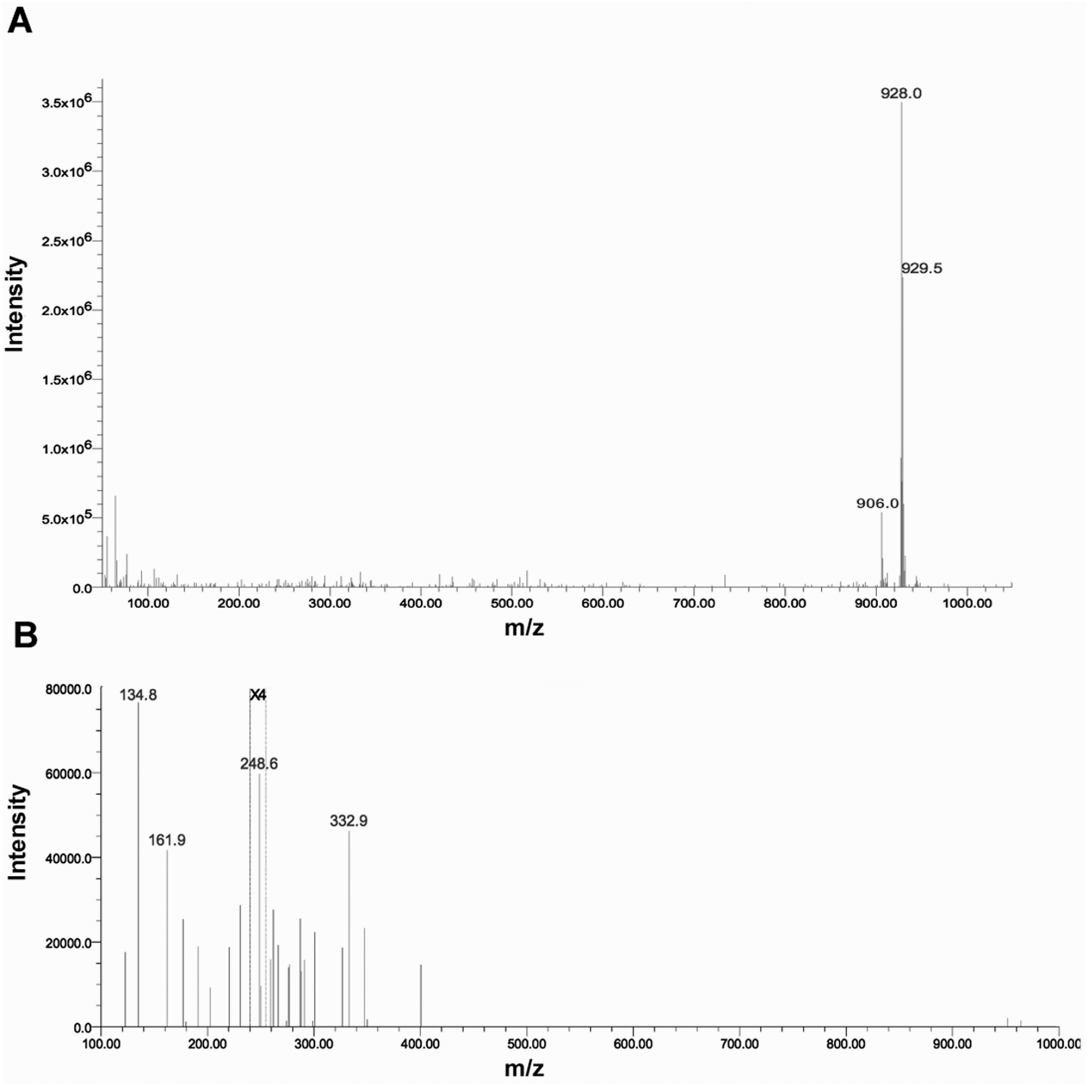
**A**. UPLC-MS (ESI+) mass spectra of TAFC standard (1 ug/mL) in acetonitrile water/0.1% formic acid. The signal at 906 m/z corresponds to the [M+H] ion mass of TAFC, and the signal at 928 m/z corresponds to the [M+Na] ion mass. The data show that the [M+Na] ion is the most abundant molecular ion of TAFC. **B**. MS/MS (ESI+) fragmentation spectra of the [M+Na] ion of the TAFC standard showing the two product ions, 134.8 m/z and 248.6 m/z, acquired under similar MS/MS conditions as in the test method. The spectrum was acquired using a collision energy of 60 eV. The signals between m/z 200 to 255 were enhanced 4 times (X4).

LC-MS/MS detection of TAFC was performed by positive electrospray ionization and with MRM data acquisition. At physiologically relevant concentrations, TAFC exhibits two molecular ions; the [M+H] ion at 906.0 m/z, and the [M+Na] ion mass at 928.59 m/z [32], with the [M+Na] ion being the most abundant molecular ion of TAFC. Consequently, the [M+Na] ion at 928.59 m/z was chosen for the LC-MS/MS method development. Two MRM transitions were monitored for quantitation of TAFC: [M+Na]^+^ 928.59 m/z to two main fragment ions, 134.8 m/z and 248.6 m/z. Quantification was performed by summing the MS/MS signal responses of both product ions. Fig 1 shows the UPLC-MS (ESI+) mass spectra of the TAFC standard (1 μg/mL) and the MS/MS fragmentation spectra.

**Fig 2:**
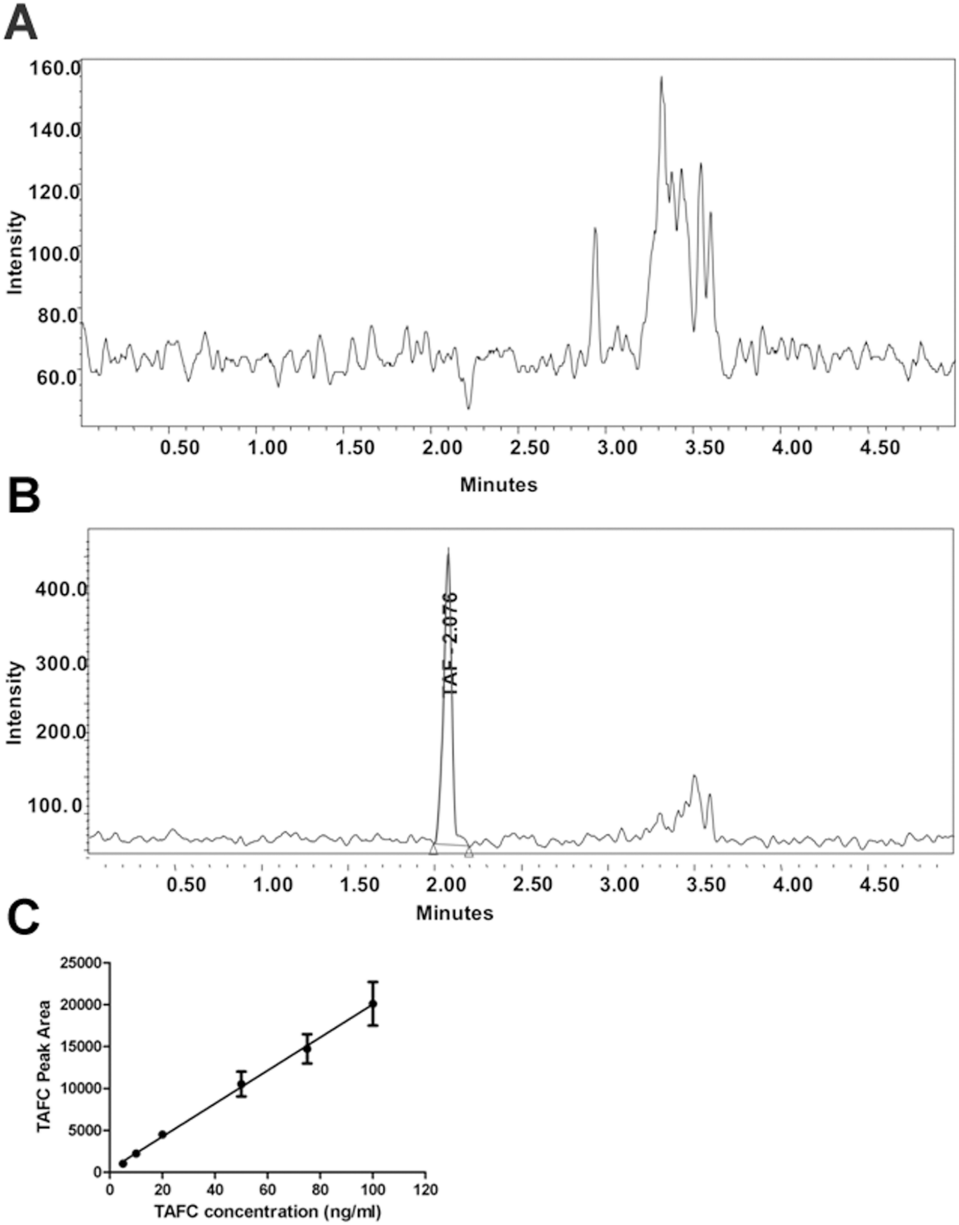
UPLC-MS/MS detection of TAFC. **A**. Chromatogram of healthy serum without the addition of TAFC. **B**. Pure TAFC (5 ng/ml) was spiked into healthy serum, extracted and detected via UPLC-MS/MS. Note the difference in magnitude of the Y-axis. **C**. Representative standard curve of TAFC over a range of 1–100 ng/ml. The values represent the mean ±SD of n = 3 samples.

Sample chromatograms of serum extracts with and without TAFC spiking are shown in Fig 2A and 2B. The limit of detection (LOD) for the instrument was found to be ≤1 ng/ml of TAFC, while the lower limit of quantitation (LLOQ) was 5 ng/ml.

A standard curve for TAFC detection was constructed and found to be linear over the range of 5 ng/ml to 1000 ng/ml with a correlation coefficient (r) of 0.999. Fig 2C shows the curve in the 1–100 ng/ml range. Furthermore, the UPLC-MS/MS method was determined to be specific for TAFC as no interfering serum matrix component co-eluted with TAFC and there was no carry-over seen when a blank sample was analyzed immediately after analysis of an extracted serum sample (Table 1). The percent deviation from theoretical values of the measured amounts of TAFC in standard solutions of TAFC in serum were less than 15% over the 5 ng/ml to 750 ng/ml quality control (QC) standards evaluated. The method was found to be accurate and precise, and has high recovery of TAFC from spiked serum samples. The accuracy (% deviation), precision (%RSD) and recovery (%) of the method at the LLOQ were ≤ ±15.0%, 3.4% and 117.1% respectively (Table 1).

**Table 1:** Evaluation of method accuracy, precision, and recovery of TAFC from spiked serum samples. All values represent the mean of three independent samples ± SD.

Concentration of TAFC spiked into QC standards, or spiked into serum ng/ml	Measured TAFC in QC standards ng/ml (%RSD)	Recovery of TAFC from serum % (%RSD)
**0**	Not detected	Not applicable
**5**	4.6 ± 0.2 (-8.7)	117.1 ± 9.7 (3.4)
**100**	111.1 ± 2.4 (11.1)	119.6 ± 17.5 (2.2)
**750**	747.2 ± 39.6 (-0.4)	120.6 ± 6.0 (5.3)

### Patient sample testing

**Fig 3:**
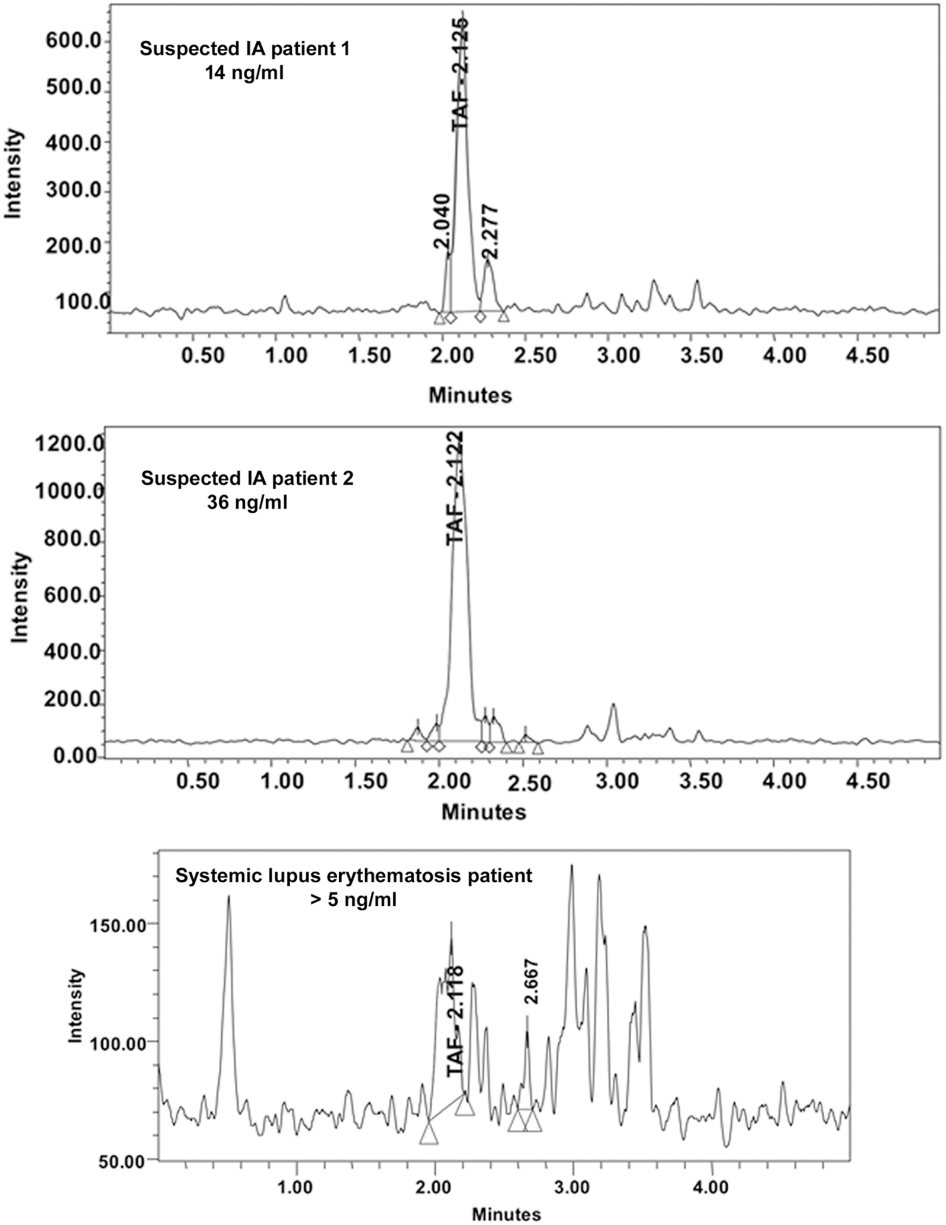
Representative TAFC chromatograms from various patient serum samples. Note the difference in scale of the Y-axis. All samples were concentrated 4-fold prior to TAFC measurement.

Human serum samples were analyzed and serum from SLE patients and healthy individuals was used to establish the background TAFC level. Representative chromatograms of patient samples are shown in Fig 3.

**Fig 4:**
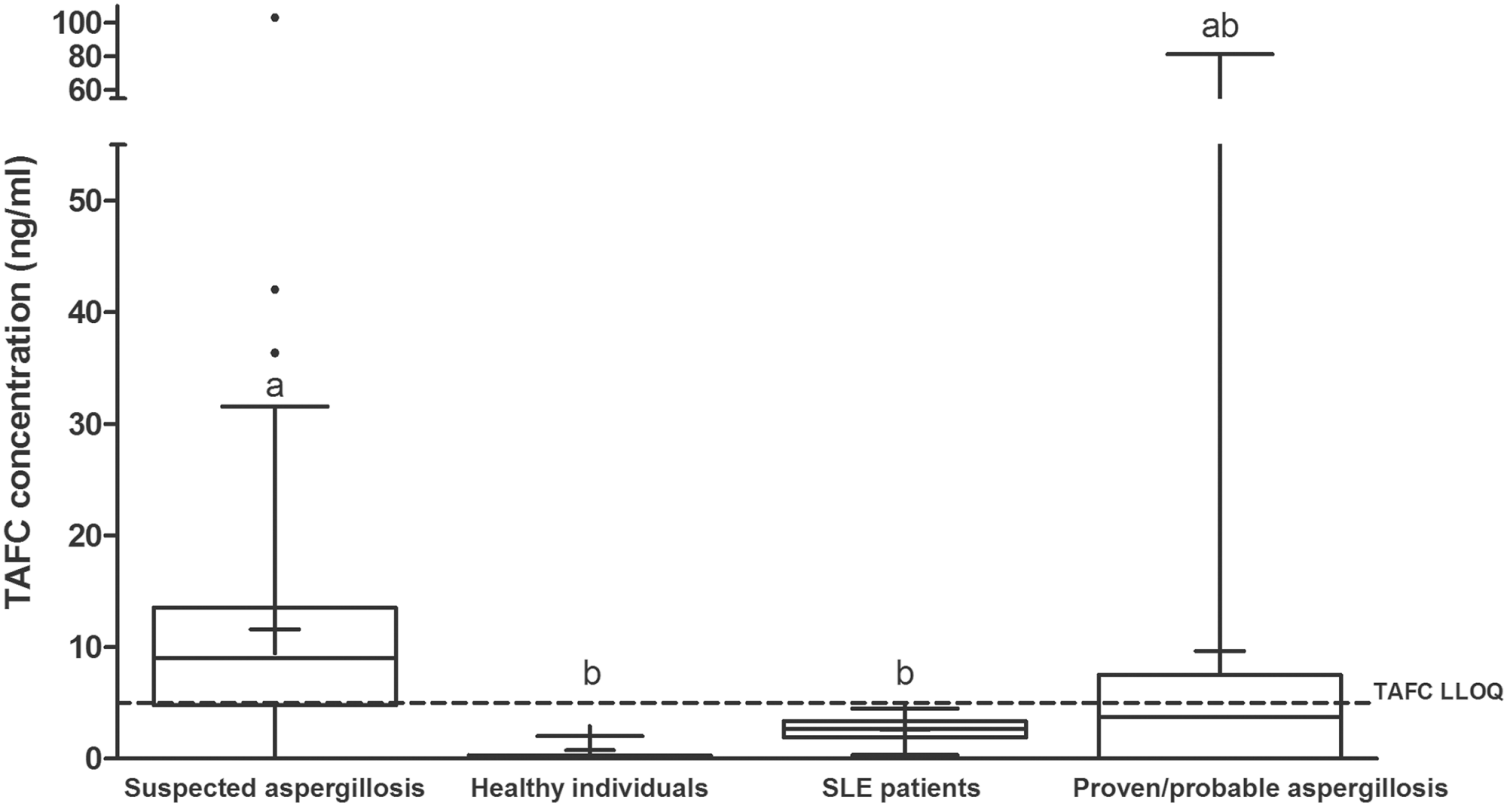
Box and whisker plots of the TAFC levels detected in healthy individuals, in patients with SLE, in patients suspected of having aspergillosis and those diagnosed with proven or probable IA. The bars represent the lower and upper limits of the data points within the 95% confidence interval. The bar in the middle of each box represents the median; outliers are shown as dots. Data are from 4-fold concentrated extracts. The LLOQ of TAFC is shown as a dotted line (5 ng/ml). Means with the same letter are not significantly different from each other (Dunn’s test, p < 0.05).

Serum from 3 healthy individuals had TAFC levels of 0.8 ± 1.1 ng/ml, well below the LLOQ of 5 ng/ml. Sera from SLE patients were also analyzed. The mean of the SLE group was slightly higher (2.6 ± 1.1 ng/ml) than the healthy patients though still below the LLOQ (Fig 4).

**Fig 5:**
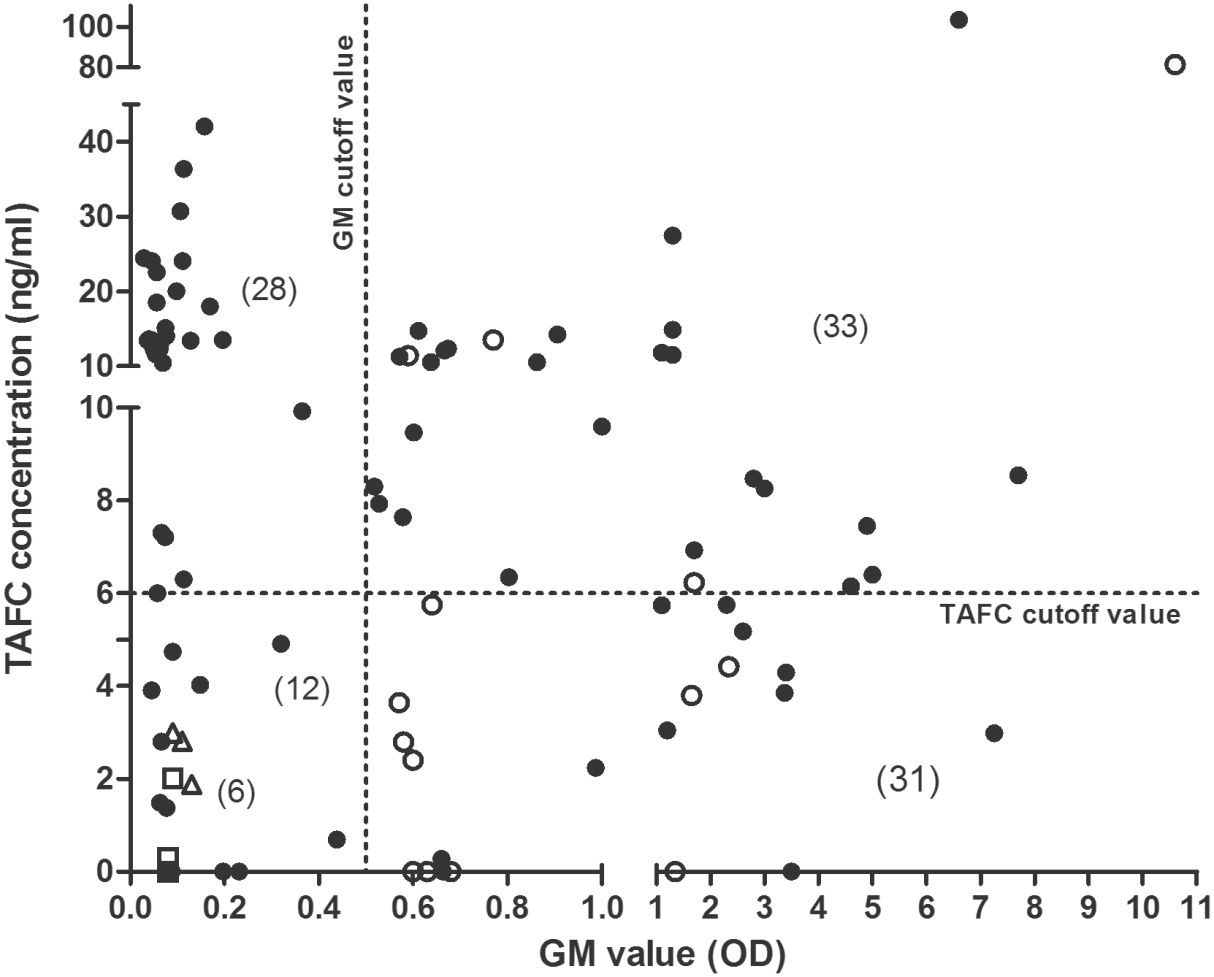
Amounts of TAFC and GM values detected in 4-fold concentrated extracts of suspected aspergillosis patient serum samples (76), 6 control patient serum samples and 14 proven or probable IA samples as analyzed by LC-MS/MS and GM testing. Of the suspected samples (black circles), 25 were TAFC positive and GM positive, 28 samples were TAFC positive and GM negative, 11 samples were TAFC negative and GM positive, and 12 were both TAFC and GM negative. All control sera (n = 6 representing 3 healthy sera (unfilled squares) and 3 sets of pooled SLE sera (unfilled triangles)) were TAFC and GM negative. Note that the 13 SLE patient samples were analyzed for TAFC individually; however, GM values were obtained from 3 pooled samples; therefore, the TAFC values were also averaged in these pools. Four of the proven or probable IA samples (unfilled circles) were TAFC positive and GM positive. The axes are expanded in the lower ranges to show the data close to the threshold values.

Using the data from the SLE group, we established a cut-off value for a positive TAFC value as 3 SD above the mean SLE value, i.e., ≥6 ng/ml. The mean and median of TAFC concentration in suspected aspergillosis serum samples were 11.6 ng/ml and 9.0, respectively. Patients with proven or probable aspergillosis had mean and median TAFC levels of 9.7 ng/ml and 3.7 ng/ml, respectively (Fig 4). We compared the results of the TAFC analysis with the GM analysis for each of the samples. Of the 76 samples from patients with suspected aspergillosis, TAFC was considered positive (≥ 6 ng/ml) in 53 of the samples (70%). Of 36 GM-positive suspected aspergillosis samples (GMI≥0.5) 25 were also positive for TAFC (69%; Fig 5). An additional 28 samples were positive for TAFC but negative for GM. Only 8 samples with a GMI greater than 1.0 (n = 58) had negative TAFC values below 6 ng/ml. When serially sampled patients were excluded, TAFC was detected in 35 of 51 patients (69%); 17 of these patients were GM positive (33%).

**Fig 6:**
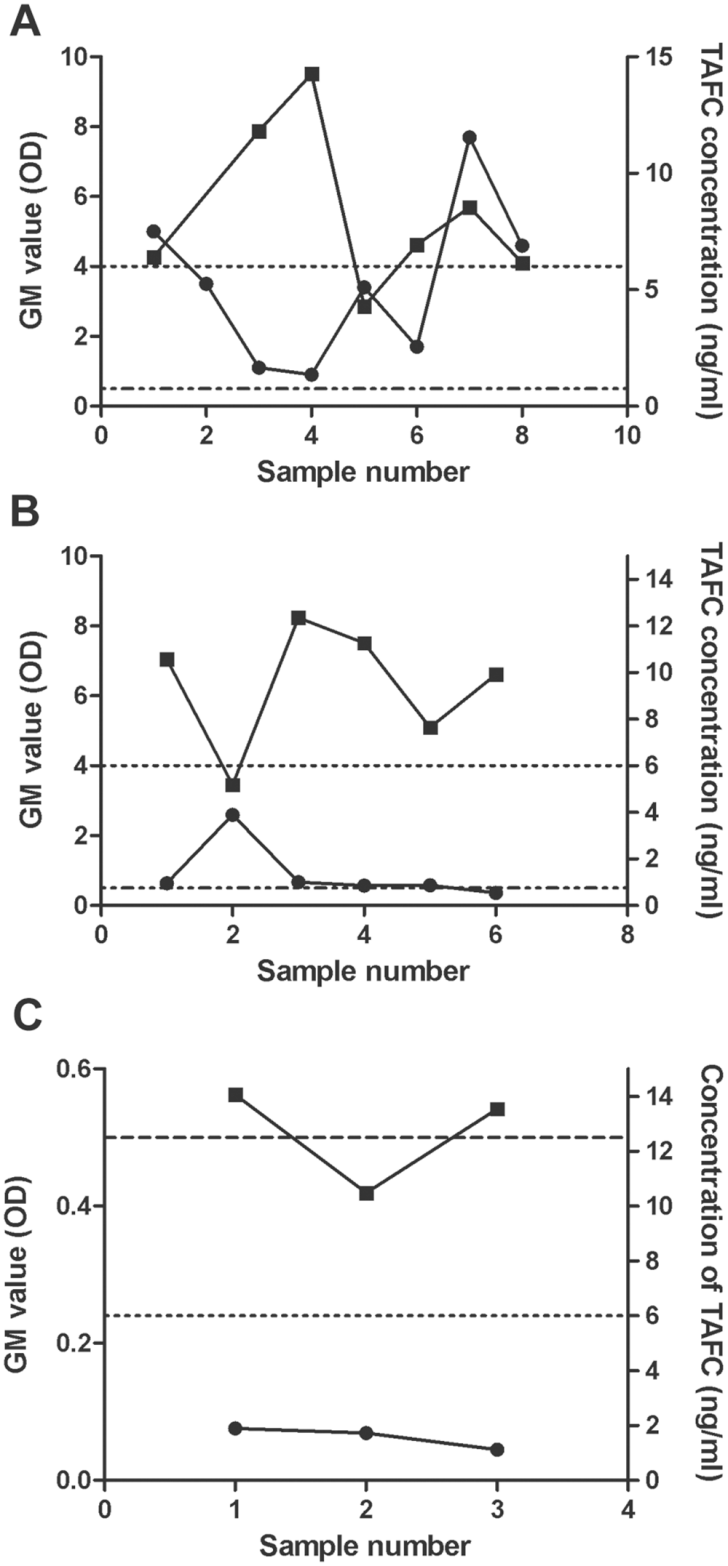
TAFC and GM values for serial samples from selected patients. TAFC (black squares) and GM (black circles) values were determined for 3 patients (**A**), (**B**) and (**C**) over time. Detection limits are noted by the dashed line (GM) and the dotted line (TAFC). The interval between the samples for each patient averaged 13 days.

Interestingly, when GM values were compared to TAFC levels in a single patient over time, the levels were inversely related (Fig 6). In another patient (Fig 6C), TAFC was positive in all 3 samples but these were negative for GM. Of the 14 proven or probable aspergillosis serum samples, TAFC was identified in 4 samples (28%).

**Fig 7:**
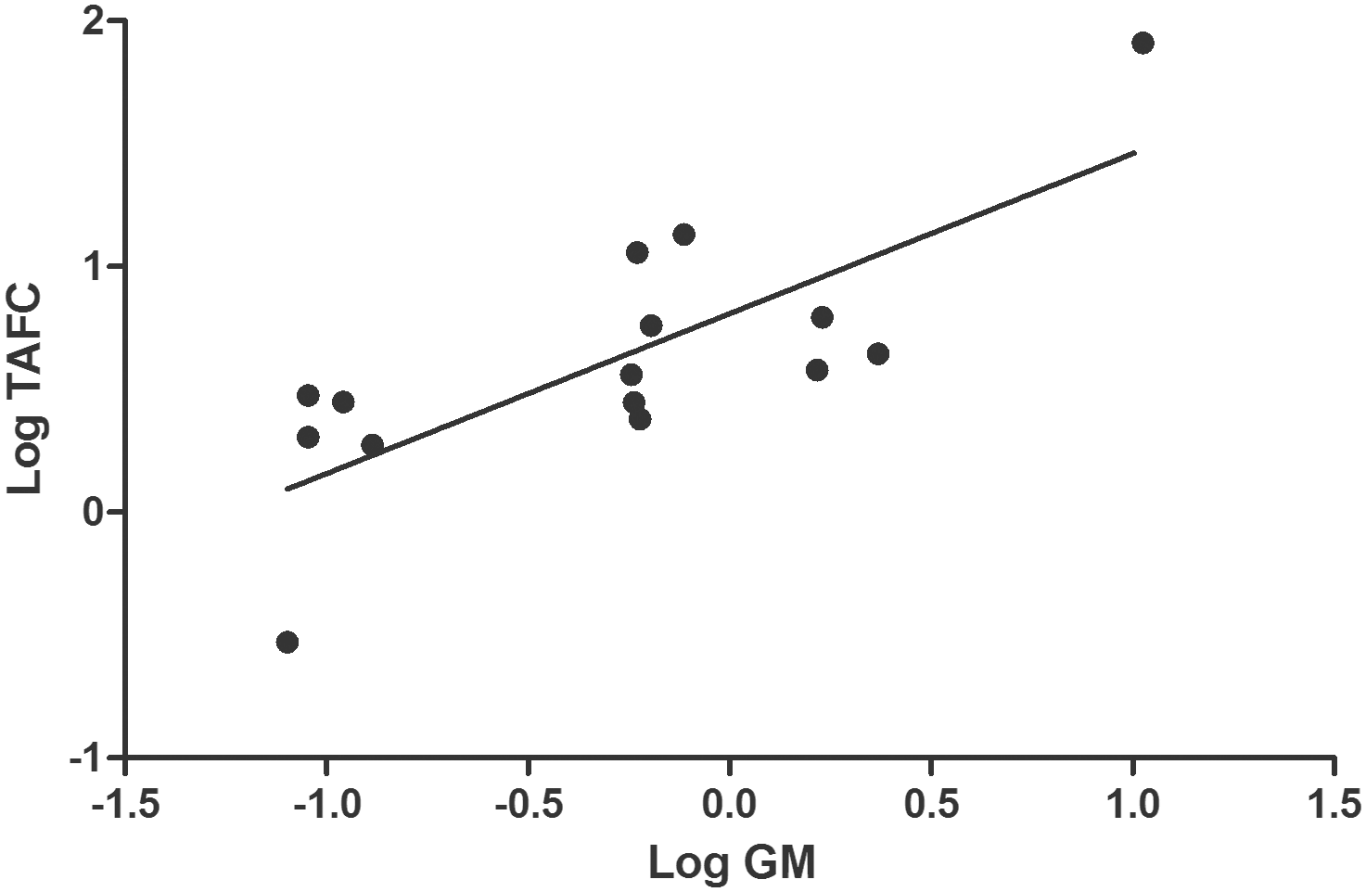
Correlation of logarithmically-transformed TAFC and GM values in sera from patients with proven/probable IA, healthy individuals and SLE patients (the three SLE patient samples were pooled from 13 individuals). The Pearson r value was 0.77 (95% confidence interval of 0.42 to 0.92) and a P value (two-tailed) of <0.001.

TAFC and GM values of sera from patients with known health status (those with proven/probable IA, healthy individuals and SLE patients) were further analyzed. TAFC and GM values were logarithmically transformed for normality, and a statistically significant correlation was found with a Pearson r value of 0.77 (95% confidence interval of 0.42 to 0.92) (Fig 7).

## Discussion

An LC-MS/MS method was developed for detecting the fungal siderophore, *N*,*N'*,*N"*-triacetylfusarinine C (TAFC) in serum from patients at risk of developing invasive aspergillosis. Evaluation of the method using control serum spiked with known amounts of TAFC revealed that the method was precise with a high recovery of TAFC and upper and lower limits of quantitation were 750 ng/ml and 5 ng/ml, respectively. The limit of detection (LOD) for TAFC was ≤1ng/ml; this is in agreement with detection of other circulating serum proteins[38]. Analysis of healthy sera (n = 3) and SLE patient sera (n = 13) indicated TAFC values less than the LLOQ. In contrast, of the 76 serum samples from patients who were suspected of having IA, 53 had levels of TAFC above the cut-off value (≥6 ng/ml). GM values were positive in 36 of these samples, and 25/36 (69%) were also TAFC positive by LC-MS/MS analysis. Of the 14 samples from patients with proven/ probable aspergillosis, 4 were found to be TAFC positive (28%). The lower proportion of TAFC-positive samples in the proven/probable IA patients compared to the suspected IA group may reflect a higher use of antifungal drugs in the former group.

TAFC is a potential biomarker for early diagnosis of IA because it is secreted during the germination phase of fungal growth and it is chemically stable in serum. We have previously shown that micromolar levels of TAFC accumulate in vitro if *A*. *fumigatus* conidia (10^6^/ml) are germinated in medium containing human serum[33]. The cluster of TAFC—positive samples in the suspected IA group that were GM negative may represent early stages of IA; however, this cannot be confirmed without a prospective study. Secretion of TAFC in vivo in later stages of infection may decrease as other iron sources become available to the fungus (e.g., proteolytic release of iron from host proteins[32]) at which point other markers such as GM may become more useful. In a retrospective study on hematopoietic stem cell transplant recipients, Nguyen et al. (2011) found that GM tests done using bronchoalveolar fluid (BALF) showed a greater test sensitivity compared to tests done on serum[39]. Hence, it would be useful to compare GM and TAFC levels in BALF in patients with proven/probable invasive aspergillosis.

Diagnosis of IA requires clinical, radiographic, histologic, and microbiologic evidence. Weaknesses in all of these facets make the diagnosis difficult. Detection of the cell wall-derived compounds, galactomannan (GM) and (1→3)-β-D-glucan (BG) are used in IA diagnosis. BG assay detects circulating levels of BG in serum samples of patients suspected to have IA. For the Fungitell assay (Assoc. of Cape Cod Inc., USA), a BG level of ≥ 80 pg/ml is considered positive for IA. However, the BG assay is not exclusively indicative of invasive aspergillosis as BG is also present in the cell wall of other pathogenic fungi and yeasts including, *Candida*, *Fusarium*, and *Pneumocystis* species[40–42]. In addition to *A*. *fumigatus*, TAFC is also produced by the pathogen, *Aspergillus nidulans*[43]. Detection of TAFC would be useful for diagnosis of both *A*. *fumigatus* and *A*. *nidulans* infections in hematology patients; however, a negative test would not rule out invasive mycoses by other mould pathogens such as *Scedosporium*, *Fusarium* or members of the Mucorales order that do not secrete TAFC.

Polymerase chain reaction (PCR) has also been proposed as a diagnostic test for IA, based on amplification and detection of *Aspergillus*-specific DNA. Typically, the 28S, 18S or inter-transcribed (ITS) ribosomal gene regions are targeted for amplification. These assays are sensitive and have been reported to detect as little as five to six conidia per ml of spiked blood[19,44]. When using qPCR to amplify the ITS region from blood samples, the sensitivity and specificity range from 24–80% and 57–100%, respectively[19]. Using nested qPCR to detect the 28S rRNA gene, sensitivity and specificity ranged from 69–95% and 36–73%, respectively[18,19]. Results vary for qPCR results from BAL samples; Hoenigl et al., (2014) report sensitivity and specificity data of 70% and 100%, respectively, when amplifying the 18S rRNA gene, while Buess et al., (2012) obtained 0% sensitivity and 71% specificity when amplifying the same gene region[28,45]. While attempts have been made to standardize DNA extraction from whole blood and for qPCR conditions in general[29,46], qPCR results remain highly variable and optimal DNA extraction efficiency requires large volumes (≥ 3 ml), which are sometimes not possible to obtain. For these reasons, PCR is not yet used in the diagnostic criteria for invasive aspergillosis outlined by the EORTC[31,47]. For our study, we used only 200 μL of serum for the TAFC assay and assays with spiked sera showed that the precision of the method using this volume was high. It may also be possible to detect TAFC in other body fluids; for example, rapid clearance of ^68^Ga-labelled TAFC has shown that the compound accumulates in urine[48]. Similarly, antigens containing β-(1,5) galactofuranose residues, present in the serum of guinea pigs infected with *A*. *fumigatus*, rapidly localized to the bladder and were detectable in urine via immunoassays[24].

The use of analytical methods, such as LC-MS/MS as diagnostic tools has many advantages over traditional molecular techniques, including high throughput capabilities, higher sensitivity, particularly for low molecular weight compounds such as siderophores, and the ease of standardization. For these reasons, the use of this technology in clinical applications has increased dramatically in recent years and LC-MS/MS is now routinely used to measure a diverse range of targets including drugs and toxins and more recently, hormones[49,50]. LC-MS/MS analysis has been used to detect other fungal-specific products in patient samples. For example, LC-MS/MS was used to detect the secreted mycotoxin, gliotoxin, in the serum of individuals suspected of having invasive aspergillosis[51]. The developed assay was sensitive, with a lower limit of quantitation (LLOQ) for gliotoxin in serum of 10 ng/ml. Upon analysis of 30 patient serum samples, the levels of gliotoxin were above the LLOQ only in two serum samples and these were both negative for GM. Furthermore, the production of gliotoxin is not essential for virulence of *A*. *fumigatus* in neutropenic hosts[52]. In another study, LC-MS detection of the siderophore, *N*^*α*^-methyl coprogen B in sputum samples from cystic fibrosis patients was shown to be indicative of infection with *Scedosporium apiospermum*; however, quantification limits have not been established for this assay[53]. Other attempts have been made to develop assays based on detection of fusarinine C in biological samples[54]; in this study, the detection limit was 3 μg/ml in guinea pig urine and serum.

We have shown that the secreted fungal siderophore TAFC can be detected in serum samples from patients at risk for developing invasive aspergillosis using LC/MS/MS with limits of quantitation of 5 ng/ml using only 200 μL of serum. These data warrant a prospective study of patients to determine whether this method provides improved detection of IA, particularly during the early stages of infection.
