# Correction: Detection of a Serum Siderophore by LC-MS/ MS as a Potential Biomarker of Invasive Aspergillosis

**DOI:** 10.1371/journal.pone.0155451

**Published:** 2016-05-10

**Authors:** Cassandra S. Carroll, Lawrence N. Amankwa, Linda J. Pinto, Jeffrey D. Fuller, Margo M. Moore

In Fig 3, the TAFC value for the SLE patient data is incorrect. The correct TAFC value is <5 ng/ml. Please see the corrected Fig 3 here.

**Fig 3:**
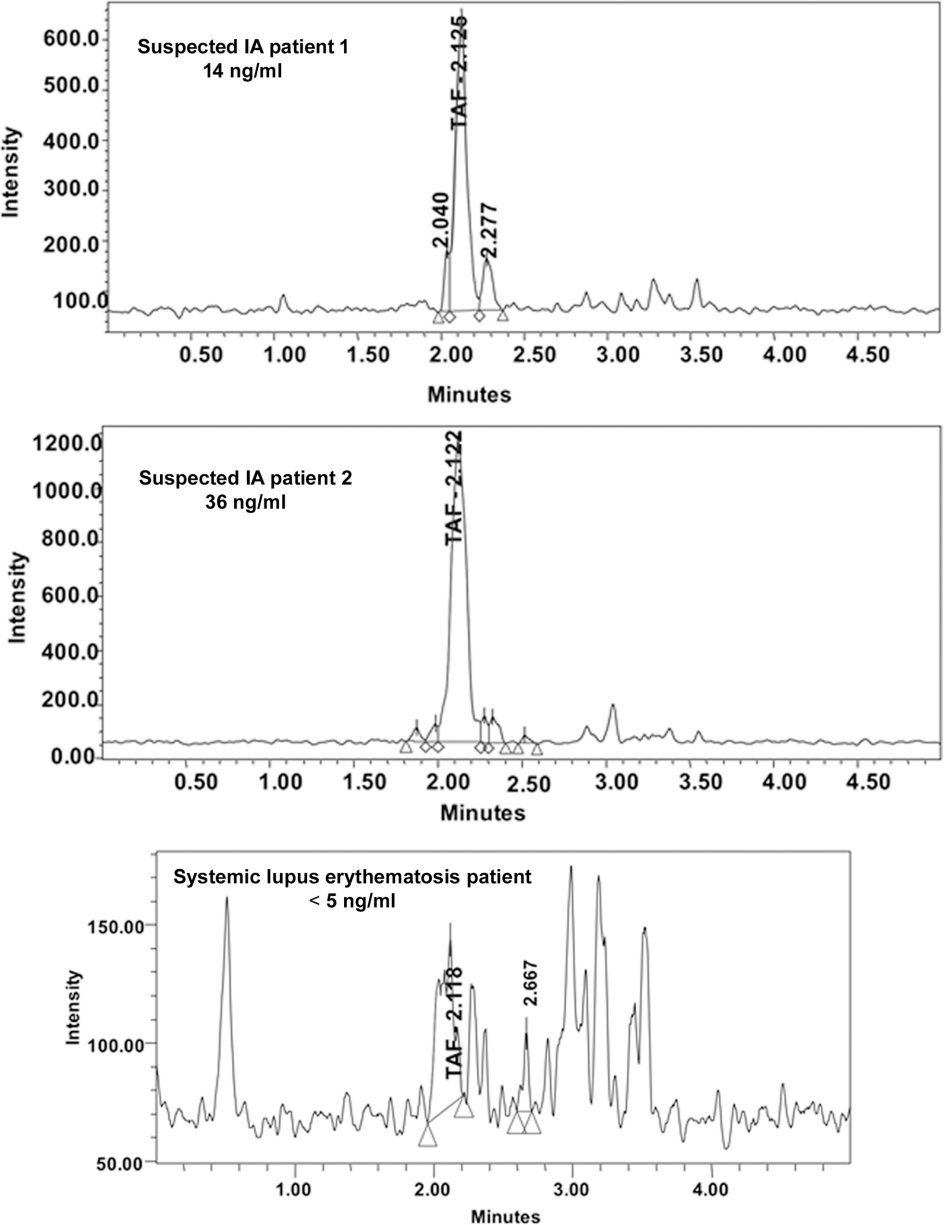
Representative TAFC chromatograms from various patient serum samples. Note the difference in scale of the Y-axis. All samples were concentrated 4-fold prior to TAFC measurement.
